# Using Functional Near-Infrared Spectroscopy to Assess Brain Activation Evoked by Guilt and Shame

**DOI:** 10.3389/fnhum.2020.00197

**Published:** 2020-06-10

**Authors:** Lian Duan, Qiudi Feng, Pengfei Xu

**Affiliations:** ^1^Shenzhen Key Laboratory of Affective and Social Neuroscience, Center for Brain Disorders and Cognitive Sciences, Shenzhen University, Shenzhen, China; ^2^School of Policing Studies, Shanghai University of Political Science and Law, Shanghai, China; ^3^Center for Emotion and Brain, Shenzhen Institute of Neuroscience, Shenzhen, China; ^4^Great Bay Neuroscience and Technology Research Institute (Hong Kong), Hong Kong, China

**Keywords:** functional near-infrared spectroscopy, guilt, shame, social cognition, moral emotion

## Abstract

Functional near-infrared spectroscopy (fNIRS) is a promising brain imaging modality for studying the neural substrates of moral emotions. However, the feasibility of using fNIRS to measure moral emotions has not been established. In the present study, we used fNIRS to detect the brain activation evoked by two typical moral emotions—guilt and shame. We presented the participants with guilt and shame context to evoke emotional responses and measured the brain activity by using fNIRS. The univariate general linear model analysis showed significant activations for both emotions in the orbitofrontal cortex, dorsolateral prefrontal cortex, and middle temporal gyrus, and specific activation for guilt in the right temporoparietal junction. The multivariate classification analysis showed an overall recognition accuracy of 52.50%, which was significantly higher than the chance level in classifying the guilt, shame, and neutral emotions. These results suggested the feasibility of using fNIRS to assess the brain activation evoked by guilt and shame and demonstrated the potentials of fNIRS in studying the neural correlates of moral emotions.

## Introduction

Moral emotions play an important role in maintaining social norms, repairing social attachments, and encouraging prosocial behaviors (Haidt, 2003; Tangney et al., 2011). Compared with those “basic” emotions (e.g., anger, happiness, sadness, disgust and fear), moral emotions (e.g., guilt and shame) support more complex social and moral functions (Wagner et al., 2011; Bastin et al., 2016). Impairment of moral emotions can lead to various psychological and behavioral disorders (Tangney et al., 1992; Kim et al., 2011). Studying the neural mechanism of moral emotions has become a focused area of cognitive neuroscience (Zhu et al., 2019).

Functional near-infrared spectroscopy (fNIRS) is a fast-developing neuroimaging technology. Functional NIRS monitors the absorption of the near-infrared light that transports through the outer cerebral cortex. The light intensity signal can be converted to the concentration change of the oxyhemoglobin (HbO) and deoxyhemoglobin (HbR), which reflect the brain’s activity (Boas et al., 2004). Functional NIRS has not only acceptable temporal and spatial resolution, but also many unique advantages such as portable, comfortable, and insensitive to head motion, providing friendly experimental environment to enhance the ecological validity of the study (Cutini et al., 2012). Functional NIRS is also very suitable for studying specific participant groups such as infants (Homae et al., 2010; Nakato et al., 2011), children (Hoshi, 2002; Perlman et al., 2014), and patients with psychiatric disorders (Irani et al., 2007). Functional NIRS also has the potential to support concurrent scan of multiple participants [i.e., hyperscanning (Babiloni and Astolfi, 2014)]. These features make fNIRS a promising modality for studying the neural underpinnings of moral emotions.

However, to our knowledge, very few studies have employed fNIRS to study moral emotions. The feasibility of applying fNIRS to study moral emotions has not been established. In the present study, we used guilt and shame as examples to validate studying moral emotions by using fNIRS. Guilt and shame are two typical moral emotions, which have been extensively studied with functional magnetic resonance imaging (fMRI) (Takahashi et al., 2004; Finger et al., 2006; Moll et al., 2007; Wagner et al., 2011; Michl et al., 2012; Roth et al., 2014; Bastin et al., 2016; Zhu et al., 2019). We elicited the participants’ guilt and shame experience using moral emotional context described by sentences. Although this vignette-based recall task may be less ecologically valid than some interaction-based task (e.g., Yu et al., 2013), it is simple to implement and can steadily elicit the guilt and shame emotion (Takahashi et al., 2004; Michl et al., 2012) and therefore serves as a good benchmark. We measured the brain activity by using fNIRS and analyzed the data by using both univariate and multivariate approaches.

## Materials and Methods

### Participants and Paradigm

Forty healthy college students (20.0 ± 2.1 years of age, 21 males and 19 females) from Shenzhen University participated in this fNIRS study. All the participants are right-handed, with normal or corrected-to-normal vision, and without any history of psychiatric or neurological disorders. All the participants gave written informed consent in accordance with the Declaration of Helsinki before the experiment. The study protocol was approved by the institutional review board at Shenzhen Key Laboratory of Affective and Social Cognitive Science, Shenzhen University.

Following the previous fMRI studies (Takahashi et al., 2004; Michl et al., 2012), the present study adopted the short sentences carrying guilt and shame information to elicit the participants’ moral emotions. We used three categories (guilt, shame, and neutral) of short Chinese sentences, and each category had 30 sentences. All the sentences were in the past tense and in the first person (see Supplementary Material). Before the experiment, we asked another group of 31 volunteers (11 males and 20 females, 18.6 ± 1.1 years of age, healthy college students) to evaluate all the 90 sentences by rating how guilty and how ashamed they felt during the situation described by each sentence using a six-point scale (0 = don’t feel guilty/ashamed at all, 5 = feel guilty/ashamed very much).

**FIGURE 1:**
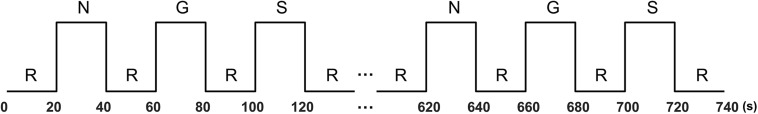
Block design paradigm. R, rest; N, neutral; G, guilt; S, shame.

The stimuli were presented in a block design. Each emotion condition consists of six 20-s blocks. In each block, five different sentences (each lasting for 4 s) of the same category were visually presented in sequence. The blocks of the three conditions were presented alternately in neutral–guilt–shame order, interleaved with a 20-s rest block (Takahashi et al., 2004; Michl et al., 2012). In the rest blocks, a fixation cross was presented in the center of the screen. There was also a rest block at the beginning and the end of the experiment (Figure 1). The stimuli were presented with E-Prime 2.0 software (Pittsburgh, PA, United States). The participants were instructed to read the sentence of each block and imagine how they feel in the described situation. After the experiment, participants also rated how guilty and how ashamed they felt for each of the sentences using a six-point scale.

### Data Acquisition and Preprocessing

**FIGURE 2:**
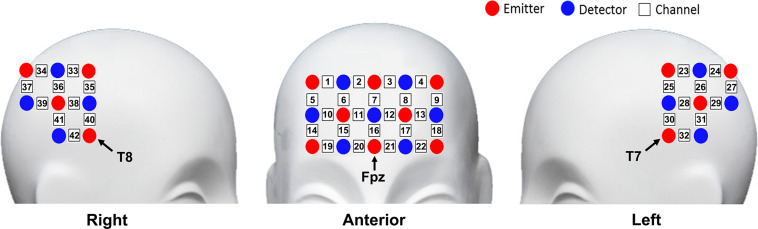
Schematic representation of optodes and channels.

The fNIRS measurement was conducted using the NIRScout continuous wave fNIRS system (NIRx Medical Technologies, New York, NY, United States). Three pieces of probe sets were used in this study. One piece was placed on the frontal area, and the other two pieces were placed on the bilateral temporal–parietal areas, forming 42 channels in total. The frontal probe set was placed by approximately putting its bottom middle optode on Fpz of the International 10-20 System (Jasper, 1958), and the bilateral temporal–parietal probe sets were placed by approximately putting their anterior inferior optode on T7 and T8, respectively (Figure 2). The source-detector distance was 30 mm. The cortex localization of the optodes and channels was obtained by using the NIRSite software (NIRx Medical Technologies) and the NIRS-SPM software (Singh et al., 2005; Ye et al., 2009).

The absorptions of the near-infrared lights at two wavelengths (785 and 830 nm) were measured with a sampling rate of 7.8125 Hz. The oxygenated (HbO) and the deoxygenated (HbR) signals were calculated with the modified Beer–Lambert law (Cope and Delpy, 1988), using differential pathlength factor of 7.25 and 6.38 for 785 and 830 nm, respectively (Hiraoka et al., 1993). The signals were 0.01–0.2 Hz bandpass filtered to remove the low-frequency drift and the high-frequency heart rate, respiration, and apparatus thermal noise (Zhang et al., 2017).

### Data Analysis

The general linear model (GLM) approach was used to calculate the brain activation map of guilt and shame. The regressors of GLM were made by convolving the block design of each condition with the canonical hemodynamic response function. Then, the model parameters of every condition were estimated channel-by-channel for all the participants. To calculate the effects of the guilt and the shame condition, contrasts that guilt minus neutral (G – N) and shame minus neutral (S – N) were constructed, respectively. The group-level analysis based on the mixed-effects model was derived by conducting a one-sample *t* test on all individual contrasts channel-by-channel to obtain the *t* statistic map (Holmes and Friston, 1998; Lu et al., 2010). Moreover, to further explore the difference between the brain activation of guilt and shame, the group-level *t* statistic maps of contrast that guilt minus shame (G − S) and shame minus guilt (S − G) were also calculated. In the present study, we mainly focused on the HbO data because of its high signal-to-noise ratio.

In addition to the univariate activation analysis, we also conducted a multivariate pattern classification analysis to explore the feasibility of recognizing the guilt and shame emotion from fNIRS signals. We pooled all the blocks of all the participants together and obtained 720 blocks, in which there were 240 guilt blocks, 240 shame blocks, and 240 neutral blocks. We then trained a simple linear supporting vector machine (SVM) classifier to classify a block belongs to which of the three categories (guilt, shame, and neutral). We used the average activation pattern of each block as the feature. Specifically, the temporally averaged time course of every channel in each 20-s task block and its pre-posed rest block were subtracted, forming a 42-channel spatial map, which was used as the feature. The classifier was validated using a 10-fold cross-validation method. To test the statistical significance of the accuracy of the classification, we conducted permutation test by randomly permuting the labels of all the blocks. We performed 10,000 times of permutation and calculated the significance of the classification accuracy. Moreover, we also used the bootstrap approach (Greening and Mitchell, 2015) to determine which channels significantly contributed to the classification. Specifically, we performed 1,000 times of independent bootstrap sampling with replacement. Then we trained 1,000 linear SVM classifiers and estimated the 99% confidence interval of every channel’s weight in classifying each of the three emotion categories. Those channels whose 99% confidence interval was either entirely above or below zero were determined as the significantly contributing channels. All the analysis was programmed with MATLAB R2019b (MathWorks Inc., Natick, MA, United States).

## Results

### Rating Results

To validate the stimulus materials, before the experiment, other volunteers than the participants engaged in the fNIRS study rated all the 90 sentences using a six-point scale (0 = don’t feel guilty/ashamed at all, 5 = feel guilty/ashamed very much). The mean ratings of guilt and shame for neutral sentences were, respectively, 0.22 (SD = 0.35) and 0.19 (SD = 0.36), for guilt sentences 3.48 (SD = 0.67) and 1.54 (SD = 0.53), and for shame sentences 2.67 (SD = 0.88) and 3.40 (SD = 0.62). The guilt sentences received higher ratings of guilt than shame (*p* = 1.17 × 10^–6^, Wilcoxon signed rank test), and the shame sentences received higher ratings of shame than guilt (*p* = 4.58 × 10^–5^). The guilt sentences received higher ratings of guilt than the neutral sentences (*p* = 1.17 × 10^–6^), and the shame sentences received higher ratings of shame than the neutral sentences (*p* = 1.17 × 10^–6^).

After the fNIRS scanning, the participants also rated the sentences. The mean ratings of guilt and shame for neutral sentences were, respectively, 0.19 (SD = 0.32) and 0.17 (SD = 0.32), for guilt sentences 3.48 (SD = 0.63) and 1.66 (SD = 0.72), and for shame sentences 2.51 (SD = 0.89) and 3.37 (SD = 0.66). The guilt sentences received higher ratings of guilt than shame (*p* = 3.85 × 10^–8^), and the shame sentences received higher ratings of shame than guilt (*p* = 1.06 × 10^–6^). The guilt sentences received higher ratings of guilt than the neutral sentences (*p* = 3.56 × 10^–8^), and the shame sentences received higher ratings of shame than the neutral sentences (*p* = 3.55 × 10^–8^). Moreover, we also calculated the participants’ accuracy in discriminating guilt from shame and *vice versa* by using both an absolute criterion and a relative criterion. Under the absolute criterion, to count as an accurate response, the participants would need to rate 3 or higher on the “shame” scale and 2 or lower on the “guilt” scale for a shame sentence, and *vice versa* for a guilt sentence. Under the relative criterion, the participants would need to rate higher on the “shame” scale than the “guilt” scale for a shame sentence, and *vice versa* for a guilt sentence, to count as an accurate response. The mean absolute and relative accuracies of all the participants for guilt were 0.59 (SD = 0.15) and 0.75 (SD = 0.18), respectively, and those for shame were 0.40 (SD = 0.15) and 0.75 (SD = 0.10), respectively. The individual-level accuracy results were listed in Supplementary Table S1.

### Activation Results

**FIGURE 3:**
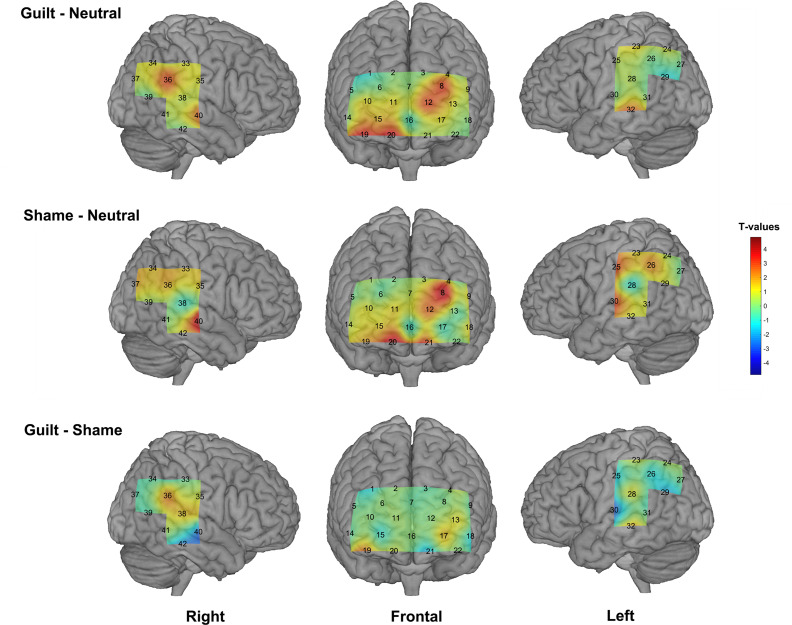
The group-level activation *t* maps derived from the HbO signal. The rows show different conditions (**top:** guilt minus neutral, **middle:** shame minus neutral, **bottom:** guilt minus shame), and the columns show different views. The activation maps were projected on the brain surface according to the cortex localization of the channels and optodes.

We found significant activation derived from the HbO signal in both guilt and shame conditions relative to neutral condition. The guilt condition relative to the neutral condition (G – N) showed significant activation in the left dorsolateral prefrontal cortex (dlPFC) (BA 46, channel 8), frontopolar (BA 10, channel 12), the right orbitofrontal cortex (OFC) (BA 11, channel 19 and 20), temporoparietal junction (TPJ) (BA 39, channel 36), and the bilateral middle temporal gyrus (MTG) (BA 21, channel 32 and 40) (Table 1 and Figure 3). The shame condition relative to the neutral condition (S – N) showed significant activation in the left dlPFC (BA 46, channel 8), the bilateral OFC (BA 11, channel 20 and 21), the left postcentral gyrus (BA 2, channel 30), and the right MTG (BA 21, channel 40) (Table 1 and Figure 3). However, no significant result was found for (G – S) or (S - G) conditions.

**TABLE 1:** Brain activations for all contrasts.

	Channel	Region	BA	*t* value	Significance
G > N	8	L dorsolateral prefrontal cortex	46	3.14	*
	12	L frontopolar	10	3.02	*
	19	R orbitofrontal cortex	11	3.91	**
	20	R orbitofrontal cortex	11	4.24	**
	32	L middle temporal gyrus	21	2.92	*
	36	R temporoparietal junction	39	3.03	*
	40	R middle temporal gyrus	21	2.81	*
S > N	8	L dorsolateral prefrontal cortex	46	4.08	**
	20	R orbitofrontal cortex	11	4.84	***
	21	L orbitofrontal cortex	11	3.42	**
	30	L postcentral gyrus	2	3.29	**
	40	R middle temporal gyrus	21	4.56	***
G > S	None	–			
S > G	None	–			

* *p* < 0.05, ** *p* < 0.01, *** *p* < 0.001. All significances were FDR corrected.

### Classification Results

**FIGURE 4:**
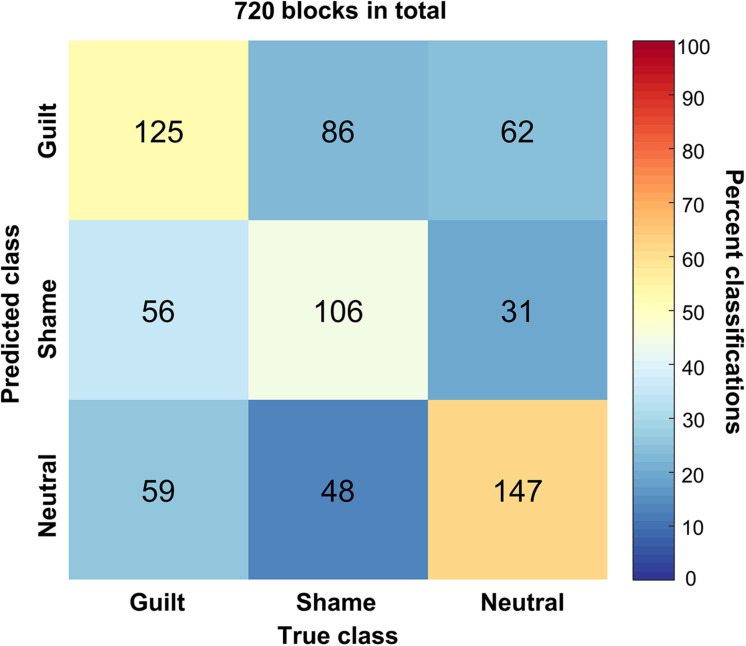
The confusion matrix of the classification. The *x* axis refers to the true categories, and the *y* axis refers to the classifier outputs. The integers in the matrix show number of samples. The color encodes the percentage of a class of blocks (*x*) classified into a predicted class (*y*).

We trained a linear SVM classifier to classify the guilt, shame, and neutral blocks and tested it using cross-validation method. Figure 4 illustrates the confusion matrix of the classification based on the HbO signal. The columns of the matrix refer to the true categories, and the rows refer to the classifier outputs. Of the 240 guilt blocks, 125 blocks were correctly classified as “guilt,” 56 blocks were classified as “shame,” and 59 blocks were classified as “neutral.” Of the 240 shame blocks, 106 blocks were correctly classified as “shame,” 86 blocks were classified as “guilt,” and 48 blocks were classified as “neutral.” Of the 240 neutral blocks, 147 blocks were correctly classified as “neutral,” 62 blocks were classified as “guilt,” and 31 blocks were classified as “shame.” The accuracy of guilt, shame, and neutral classification reached 52.08% (*p* < 1 × 10^–4^, permutation test, one-tailed), 44.17% (*p* < 0.01), and 61.25% (*p* < 1 × 10^–4^), respectively. The overall accuracy was 52.50% (*p* < 1 × 10^–4^). Across different emotions, only the neutral classification showed significantly higher accuracy than the shame classification (*p* < 0.01). The classification accuracy comparison showed no significant difference between guilt and shame (*p* = 0.14) and between neutral and guilt (*p* = 0.09).

Moreover, the bootstrap analysis determined the channels significantly contributing to the classification models (Table 2). The significant contributing channels were channels 8, 12, 19, 20, and 36 in the classification of guilt and were 8, 12, 19, and 20 in the classification of shame, which all had positive weights in the model. The significant contributing channels in the classification of neutral blocks were channels 30 and 40, which had negative weights in the model.

**TABLE 2:** Contributing channels in the classification.

	Channel	Region	BA	Weight	Confidence interval
Guilt	8	L dorsolateral prefrontal cortex	46	2.00	[0.79, 3.21]
	12	L frontopolar	10	1.83	[0.29, 3.36]
	19	R orbitofrontal cortex	11	2.41	[0.81, 4.01]
	20	R orbitofrontal cortex	11	3.00	[1.61, 4.39]
	36	R temporoparietal junction	39	2.17	[0.70, 3.64]
Shame	8	L dorsolateral prefrontal cortex	46	2.49	[1.28, 3.70]
	12	L frontopolar	10	1.95	[0.61, 3.28]
	19	R orbitofrontal cortex	11	1.39	[0.03, 2.74]
	20	R orbitofrontal cortex	11	3.18	[1.79, 4.57]
Neutral	30	L postcentral gyrus	2	-1.52	[-2.97, -0.06]
	40	R middle temporal gyrus	21	-1.81	[-3.13, -0.50]

## Discussion and Conclusion

The aim of the present study is to investigate the feasibility of using fNIRS to study two typical moral emotions—guilt and shame. We presented the participants with guilt and shame context to evoke emotional responses and measured the brain activity by using fNIRS. The subjective ratings results suggested that the sentences used in the experiment could effectively evoke the intended emotional responses of shame and guilt. On the one hand, the brain activation results revealed common brain regions involved in both guilt and shame processing, including the OFC, the dlPFC, and the MTG. Theoretically, these brain regions covered important functions involved in moral emotion provocation and processing such as social value encoding, moral judgment and decision making, cognitive control, and emotional semantic understanding. Moreover, these brain regions were also reported in fMRI studies on guilt and shame. For example, dlPFC was found activated in either guilt minus neutral condition (Wagner et al., 2011), shame minus neutral condition (Roth et al., 2014), or both conditions (Michl et al., 2012). Orbitofrontal cortex was found activated in guilt minus neutral condition (Roth et al., 2014) and was also reported to be activated by embarrassment (Takahashi et al., 2004), a moral emotion that is very similar with shame (Lewis, 1993; Tangney et al., 1996). Middle temporal gyrus was found activated in guilt minus neutral condition (Zhu et al., 2019) or both guilt minus neutral and shame/embarrassment minus neutral condition (Takahashi et al., 2004; Wagner et al., 2011; Roth et al., 2014). Being highly consistent with these previous studies, our results suggested that fNIRS can effectively capture the guilt- and shame- related brain activations.

On the other hand, although the present study did not find significant result in (G – S) condition or *vice versa*, it is worth noting that our results showed significant activation in the right TPJ specific to the (G – N) condition instead of the (S – N) condition. This result is in accord with the study of Zhu et al. (2019), which found significant activation in the right TPJ in guilt minus shame condition. Theoretically, compared with shame, guilt may involve more psychological process of understanding and empathizing the victim’s situation and mood (Tangney and Dearing, 2003; De Hooge et al., 2007; Tangney et al., 2007; Yu et al., 2013; Nelissen, 2014), whereas TPJ is implicated a critical “Theory of Mind” region supporting mentalizing, understanding, and reasoning about others’ beliefs and intentions (Bzdok et al., 2013; Schurz et al., 2014). Thus, our results suggested that fNIRS can reflect the neural difference between guilt and shame to some extent. However, our results also suggested that fNIRS may be not so sensitive as fMRI in directly detecting the relative difference between guilt and shame because of its lower signal-to-noise ratio than fMRI (Ye et al., 2009). Besides, the paradigm used in our experiment repeatedly and alternately evoked the participants’ guilt and shame experience, which might also cause fatigue and dim the difference of the feeling and neural response between guilt and shame.

In addition to the univariate activation analysis, we also conducted a multivariate classification analysis to the data. The results showed an overall accuracy of 52.50% (the chance level was 33.33% for this three-class classification problem). More specifically, the recognition accuracies of the guilt, shame, and neutral blocks reached 52.08, 44.17, and 61.25%, respectively, and were steady across different cross-validation parameters (see Supplementary Table S2). These results indicated that fNIRS is capable in distinguishing guilt and shame not only from neutral control but also from each other. Moreover, the contributing channel analysis revealed the most predictive channels in the classification of guilt, shame, and neutral. On the one hand, it could be viewed that there was an overlap between the predictive channels of guilt and shame, suggesting that guilt and shame may share some common cognitive antecedents (e.g., Maley, 2015). On the other hand, guilt showed channel 36 (rTPJ) as its unique predictive channel, which further corroborates the univariate analysis result, and emphasized the critical role of rTPJ in guilt process.

The results of the present studies are based on the oxygenated (HbO) signal. We also conducted the activation analysis using the HbR signal but did not find any significant activations in any conditions. This may due to the worse signal-to-noise ratio of HbR signal than HbO (Strangman et al., 2002; Yanagisawa et al., 2010). Therefore, the HbO signal, which is more sensitive to task response (Cheng et al., 2015), may be better for guilt and shame studies than the HbR signal.

It should be noted that, compared with fMRI, fNIRS has some disadvantages in studying guilt and shame. For example, fNIRS cannot measure the deep areas of the brain such as the insula, parahippocampal gyrus, and cingulate gyrus, which also show importance in the guilt and shame processing (Michl et al., 2012; Bastin et al., 2016). In addition, fNIRS has a limited spatial resolution and cannot localize the measurement very precisely to those fine substructures of complex brain areas, which may be engaged in different functions [e.g., to distinguish the anterior portion and the posterior portion of TPJ (Zhu et al., 2019)]. Despite these disadvantages, fNIRS has its unique advantages in some specific applications. For example, moral emotions usually arise and develop from one’s early childhood and make great influence to the whole life (Barrett et al., 1993; Whittle et al., 2016). It is of tremendous importance to study their neurodevelopment process. Compared with fMRI, fNIRS is more suitable for neurodevelopment study (Peña et al., 2003; Sugiura et al., 2011; Watanabe et al., 2017). Moreover, fNIRS can easily support hyperscanning of a group of people during social interaction in a moral emotional situation. These characteristics make fNIRS a promising alternative of fMRI for studying moral emotions.

The present study also has some limitations. First, in this feasibility study, to make the results comparable, we adopted the imagination and recall paradigm widely used in previous fMRI studies to evoke these emotions. However, this paradigm may be not able to completely reflect the essential psychological processes of guilt and shame (Bastin et al., 2016). Considering the social essence of the moral emotions, especially those components related to real-time social interactions, it could provide new perspective for viewing their neural mechanism by acquiring and analyzing the neural activity data of all the participants engaged in the social context (e.g., a guilty individual and his victim, or a shame-feeling individual and the spectator) (Konvalinka and Roepstorff, 2012). In future studies, we will extend the paradigm to daily life situations such as cooperation and face-to-face communication, which involve real social interaction, and use hyperscanning approach to scan multiple participants’ brains involved in the interaction. Second, the present study did not analyze the effect of multiple subcategories of guilt (e.g., faults and errors of conduct, harm to others, etc.) and shame (e.g., moral character problem, be humiliated, etc.). In our experiment, the stimuli of multiple subcategories of an emotion were randomly assigned to all the blocks. Thus, we could not analyze whether the classification was equally accurate for all subcategories, or whether the activation was different across multiple subcategories. Third, the present study did not analyze the gender effect and the culture difference effect. We will investigate these issues in our future studies.

In conclusion, the present study preliminarily demonstrated the feasibility of using fNIRS to investigate the neural correlates of guilt and shame. The univariate brain activation analysis based on the HbO signal produced similar results with the previous fMRI studies. The multivariate classification analysis suggested that fNIRS has great potential in moral emotion recognition. Our study provides a foundation of using fNIRS to study guilt, shame, and other moral emotions.

## Data Availability Statement

The datasets generated for this study are available on request to the corresponding author.

## Ethics Statement

All the participants gave written informed consent in accordance with the Declaration of Helsinki before the experiment. The study protocol was approved by the Institutional Review Board at Shenzhen Key Laboratory of Affective and Social Cognitive Science, Shenzhen University.

## Author Contributions

LD and QF designed the research, analyzed the data. LD and PX performed the experiments. LD drafted the work.

## Conflict of Interest

The authors declare that the research was conducted in the absence of any commercial or financial relationships that could be construed as a potential conflict of interest.
