# Supplementary material for: Using Functional Near-Infrared Spectroscopy to Assess Brain Activation Evoked by Guilt and Shame
Source: Front Hum Neurosci. 2020 Jun 10;14:197. doi: 10.3389/fnhum.2020.00197 (PMC7298148; doi:10.3389/fnhum.2020.00197)
Supplement: Supplementary file 1 [file Table_1.DOCX]

Supplementary Material

# Supplementary Data

In this part we listed all the stimuli used in the study (both the original Chinese version and an English translation). The number following each sentence labeled the sub-category of guilt and shame according to which the sentence was made.

Guilt: 1 = Faults and errors of conduct; 2 = Harm to others; 3 = Breach of ethics, commitments, rules and expectations; 4 = Responsible for bad results (including survivor guilt).

Shame: 1 = Moral character problem; 2 = Be humiliated; 3 = Make an exhibition of oneself; 4 = Inappropriate sexual proclivities; 5 = Low competence and skills.

| **Guilt** |  |
| --- | --- |
| 我不小心打碎了亲戚家的花瓶。I broke a relative's vase by accident. | 1 |
| 我弄丢了借来的自行车。I lost my borrowed bike. | 1 |
| 我在家大声唱歌吵醒了邻居。I woke up my neighbors by singing loudly at home. | 2 |
| 我没能按时完成作业。I didn't finish my homework on time. | 3 |
| 我踢球时弄伤了同学。I hurt my classmate while playing football. | 2 |
| 我在情人节忘记买礼物。I forgot to buy a gift on valentine's day. | 3 |
| 我骑电动车在路上撞到行人。I hit a pedestrian on the road with my electric bike. | 2 |
| 我呕吐时弄脏了别人的衣服。I mucked up other people's clothes when I vomited. | 1 |
| 我不小心伤害了我的朋友。I hurt my friend by accident. | 2 |
| 我控制不住消费的冲动。I couldn't control the impulse of consumption. | 3 |
| 我炒股亏了家里很多钱。I lost a lot of money in the stock market. | 4 |
| 我没能在母亲生病时照顾她。I failed to look after my mother when she was ill. | 3 |
| 我欠了朋友1万元一直没钱还。I couldn’t pay 10000 yuan back to my friend. | 3 |
| 我弄脏了姐姐新买的连衣裙。I soiled my sister's new dress. | 1 |
| 我没给队友传球，错失了机会。I missed the chance to pass the ball to my teammate. | 4 |
| 我开车不慎撞伤了一位行人。I hit a pedestrian with my car. | 2 |
| 我是地震后家中唯一的幸存者。I was the only survivor in my family from the earthquake. | 4 |
| 我带堂弟出来玩，结果他走丢了。I lost my brother when we played together. | 4 |
| 我撒谎了，父母很失望。My parents were disappointed that I had lied. | 3 |
| 我瞒着女朋友赴另个女孩的约会。I went on a date with another girl without telling my girlfriend. | 3 |
| 我中途变卦放了朋友鸽子。I stood my friend up. | 3 |
| 我跟心上人第一次约会就迟到了。I was late for my first date with my sweetheart. | 1 |
| 我发现自己错怪了室友。I realized that I had wronged about my roommate. | 2 |
| 我上大学的钱是哥哥辍学打工攒下的。My brother left school to work to save money for my college education. | 4 |
| 我打翻了女朋友的化妆品。I knocked over my girlfriend's makeup. | 1 |
| 我工作数年还没赚到钱让父母摆脱贫困。I couldn't lift my parents out of poverty after working for years. | 4 |
| 我一直找不到机会向朋友道歉。I failed to apologize to my friend. | 3 |
| 我的糟糕表现导致队友接受了惩罚。My poor performance resulted in my teammates being punished. | 4 |
| 我请远道而来的客人吃饭，之后他腹泻了。I invited my guest from far away to dinner, and then he had diarrhea. | 4 |
| 我把捐赠灾区的旧书弄丢了。I lost my old book that I was going to donated to the disaster area. | 1 |
| **Shame** |  |
| 我考试作弊被全校通报批评了。I was criticized by the whole school for cheat on an examination. | 2 |
| 我反复失误搞砸了自己的演出。I messed up my performance by making repeated mistakes. | 3 |
| 我在图书馆打电话被管理员赶出去了。The librarian kicked me out of the library for calling. | 2 |
| 我糟糕的表现收到观众的嘘声。My poor performance was booed by the audience. | 3 |
| 我总是忍不住想去看穿着暴露的异性。I couldn't help but look at scantily clad members of the opposite sex. | 4 |
| 我偷身边人的钱。I stole money from people around me. | 1 |
| 我曾经嘲笑残疾人。I used to laugh at disabled people. | 1 |
| 我在大街上打架遭到围观。I was watched fighting by a crowd in the street. | 1 |
| 我被领导当着同事的面骂了一顿。I was scolded by the leader in front of my colleagues. | 2 |
| 我做的菜没人爱吃的。No one likes my cooking. | 3 |
| 我走在大街上突然裤裆处扯破了。I was walking down the street when my crotch ripped. | 3 |
| 我未成年的时候看过黄色电影。I saw pornographic movies when I was an underage. | 4 |
| 我（男孩）小时候进过女澡堂洗澡。When I was a boy, I went to the girls' bathhouse. | 4 |
| 我上课看小说被老师抓到。My teacher caught me reading a novel in class. | 3 |
| 我欠钱不还的名声在同学间传开了。My reputation for not paying my debts spread among my classmates. | 3 |
| 我歧视女性。I discriminate against women. | 1 |
| 我不尊重老年人。I have no respect for old people. | 1 |
| 我在面对困难的时候选择了逃避。I chose to escape in the face of difficulties. | 1 |
| 我浪费食物。I wasted food. | 1 |
| 我乘坐地铁时逃票了。I cheated on the subway. | 1 |
| 我偷过很多东西。I stole a lot of things before. | 1 |
| 我没能达到别人的期望。I didn't live up to others' expectations. | 5 |
| 我没控制住饮食，又胖了10公斤。I lost control of my diet and gained 10 kg of weight. | 5 |
| 我参与了一场网络暴力行为。I participated in an act of cyberbullying. | 1 |
| 我昨晚醉酒之后行为失态。I behaved badly after getting drunk last night. | 3 |
| 我被异性发现裤子拉链没关。I was caught unzipped by the opposite sex. | 3 |
| 我在穆斯林餐厅点餐的时候询问了猪肉。I ordered pork in Muslim restaurants by mistake. | 3 |
| 我高考失利，而朋友们都去了名牌大学。I failed the college entrance exam, while my friends went to famous universities. | 5 |
| 我小时候偷看母亲洗澡。I watched my mother take a bath when I was a child. | 4 |
| 我讲了一个不合适的笑话，大家都很严肃。I told an inappropriate joke and everyone was very serious. | 3 |
| **Neutral** |  |
| 我用手机上网。I used my phone to surf the Internet. |  |
| 我周末在家打扫卫生。I cleaned my house on a weekend. |  |
| 我乘坐地铁去公园。I went to the park by subway. |  |
| 我在商场里闲逛。I hanged out in the mall. |  |
| 我在公园里放风筝。I flied a kite in the park. |  |
| 我跟同学一起打球。I played ball with my classmates. |  |
| 我在餐厅吃饭。I had a lunch in a restaurant. |  |
| 我用电脑写作业。I used the computer to do my homework. |  |
| 我假期去国外旅行。I traveled abroad on vacation. |  |
| 我周末自习备考。I studied for the exam on the weekend. |  |
| 我和父母交谈。I talked to my parents. |  |
| 我在网上购买日用品。I bought daily necessities online. |  |
| 我回忆小时候的事。I looked back on my childhood. |  |
| 我昨天吃了三顿饭。I ate three meals yesterday. |  |
| 我晚上熬夜看球赛。I stayed up late at night watching football games. |  |
| 我逛街时租了一个充电宝。I rented a charger when I went shopping. |  |
| 我早晨起来跑步。I got up in the morning to run. |  |
| 我偶尔会看看股市。I occasionally looked at the stock market. |  |
| 我买了一辆新车。I bought a new car. |  |
| 我换了新的工作。I got a new job. |  |
| 我完成了公司的任务。I completed the company's task. |  |
| 我在KTV唱了一首歌。I sang a song at the KTV. |  |
| 我和朋友看了一场电影。I watched a movie with my friends. |  |
| 我教小侄女画画。I taught my little niece to draw. |  |
| 我不喝牛奶。I didn't drink milk. |  |
| 我在教师节送了老师一束鲜花。I sent my teacher a bunch of flowers on teacher's day. |  |
| 我与母亲昨晚通了一个电话。I had a phone call with my mother last night. |  |
| 我在国庆那天看了阅兵。I watched the parade on National Day. |  |
| 我上周感冒发烧。I had a cold and fever last week. |  |
| 我昨天去医院拔了牙。I went to the hospital to have my tooth extracted yesterday. |  |

# Supplementary Tables

Table S1. Individual-level absolute and relative rating accuracy of guilt and shame

| Participant | 1 | 2 | 3 | 4 | 5 | 6 | 7 | 8 | 9 | 10 | 11 | 12 | 13 | 14 | 15 | 16 | 17 | 18 | 19 | 20 |
| --- | --- | --- | --- | --- | --- | --- | --- | --- | --- | --- | --- | --- | --- | --- | --- | --- | --- | --- | --- | --- |
| Abs. Acc. of guilt | .37 | .80 | .57 | .57 | .67 | .60 | .63 | .43 | .77 | .63 | .83 | .73 | .40 | .57 | .63 | .73 | .30 | .80 | .63 | .73 |
| Rel. Acc. of guilt | .50 | 1 | .90 | .70 | .87 | .63 | .77 | .63 | .87 | .77 | .97 | .93 | .67 | .70 | .90 | .93 | .63 | .90 | .87 | .90 |
| Abs. Acc. of shame | .47 | .07 | .33 | .27 | .27 | .63 | .60 | .37 | .37 | .50 | .37 | .50 | .53 | .40 | .33 | .37 | .20 | .27 | .40 | .67 |
| Rel. Acc. of shame | .90 | .87 | .77 | .67 | .83 | .90 | .73 | .70 | .67 | .63 | .60 | .70 | .83 | .70 | .60 | .97 | .60 | .77 | .67 | .83 |
| Participant | 21 | 22 | 23 | 24 | 25 | 26 | 27 | 28 | 29 | 30 | 31 | 32 | 33 | 34 | 35 | 36 | 37 | 38 | 39 | 40 |
| Abs. Acc. of guilt | .37 | .50 | .63 | .37 | .53 | .73 | .57 | .50 | .47 | .60 | .53 | .73 | .57 | .83 | .63 | .30 | .73 | .63 | .67 | .23 |
| Rel. Acc. of guilt | .43 | .57 | .90 | .50 | .57 | .93 | .57 | .57 | .50 | .63 | .77 | .93 | .90 | .97 | .87 | .63 | .87 | .77 | .83 | .77 |
| Abs. Acc. of shame | .53 | .17 | .43 | .47 | .47 | .50 | .57 | .27 | .50 | .27 | .33 | .60 | .33 | .07 | .60 | .60 | .20 | .30 | .47 | .50 |
| Rel. Acc. of shame | .70 | .70 | .90 | .90 | .87 | .70 | .77 | .70 | .83 | .70 | .73 | .73 | .77 | .60 | .73 | .83 | .73 | .57 | .80 | .63 |

Table S2. Classification accuracy of different cross-validation methods

| Cross validation method | Classification accuracy (%) | | | |
| --- | --- | --- | --- | --- |
|  | Guilt | Shame | Neutral | Overall |
| 20-Fold | 50.42 | 45.00 | 60.83 | 52.08 |
| 10-Fold | 52.08 | 44.17 | 61.25 | 52.50 |
| 5-Fold | 49.17 | 45.83 | 62.08 | 52.36 |
| Leave-one-out | 49.58 | 45.83 | 58.75 | 51.39 |
